# The Negative Effect of Protein Phosphatase Z1 Deletion on the Oxidative Stress Tolerance of *Candida albicans* Is Synergistic with Betamethasone Exposure

**DOI:** 10.3390/jof7070540

**Published:** 2021-07-06

**Authors:** Ágnes Jakab, Tamás Emri, Kinga Csillag, Anita Szabó, Fruzsina Nagy, Edina Baranyai, Zsófi Sajtos, Dóra Géczi, Károly Antal, Renátó Kovács, Krisztina Szabó, Viktor Dombrádi, István Pócsi

**Affiliations:** 1 Department of Molecular Biotechnology and Microbiology, Faculty of Science and Technology, University of Debrecen, 4032 Debrecen, Hungary; emri.tamas@science.unideb.hu (T.E.); csillag.kinga@science.unideb.hu (K.C.); szaboanita991@gmail.com (A.S.); g.dora@med.unideb.hu (D.G.); pocsiistvan@unideb.hu (I.P.); 2 Department of Medical Microbiology, Faculty of Medicine, University of Debrecen, 4032 Debrecen, Hungary; nagy.fruzsina@med.unideb.hu (F.N.); kovacs.renato@med.unideb.hu (R.K.); 3 Agilent Atomic Spectroscopy Partner Laboratory, Department of Inorganic and Analytical Chemistry, University of Debrecen, 4032 Debrecen, Hungary; baranyai.edina@science.unideb.hu (E.B.); sajtos.zsofi@science.unideb.hu (Z.S.); 4 Department of Zoology, Faculty of Sciences, Eszterházy Károly University, 3300 Eger, Hungary; antalk2@gmail.com; 5 Faculty of Pharmacy, University of Debrecen, 4032 Debrecen, Hungary; 6 Department of Medical Chemistry, Faculty of Medicine, University of Debrecen, 4032 Debrecen, Hungary; szabo.krisztina05@gmail.com (K.S.); dombradi@med.unideb.hu (V.D.)

**Keywords:** antifungal therapy, *Candida albicans*, combinatorial stress, glucocorticoid, menadione, oxidative stress, protein phosphatase Z1, stress response, transcriptome analysis

## Abstract

The glucocorticoid betamethasone (BM) has potent anti-inflammatory and immunosuppressive effects; however, it increases the susceptibility of patients to superficial *Candida* infections. Previously we found that this disadvantageous side effect can be counteracted by menadione sodium bisulfite (MSB) induced oxidative stress treatment. The fungus specific protein phosphatase Z1 (CaPpz1) has a pivotal role in oxidative stress response of *Candida albicans* and was proposed as a potential antifungal drug target. The aim of this study was to investigate the combined effects of *CaPPZ1* gene deletion and MSB treatment in BM pre-treated *C. albicans* cultures. We found that the combined treatment increased redox imbalance, enhanced the specific activities of antioxidant enzymes, and reduced the growth in cappz1 mutant (KO) strain. RNASeq data demonstrated that the presence of BM markedly elevated the number of differentially expressed genes in the MSB treated KO cultures. Accumulation of reactive oxygen species, increased iron content and fatty acid oxidation, as well as the inhibiting ergosterol biosynthesis and RNA metabolic processes explain, at least in part, the fungistatic effect caused by the combined stress exposure. We suggest that the synergism between MSB treatment and CaPpz1 inhibition could be considered in developing of a novel combinatorial antifungal strategy accompanying steroid therapy.

## 1. Introduction

Betamethasone (BM) is an effective anti-inflammatory, immunosuppressive, vasoconstriction and antiproliferative agent widely used in various medical treatments [1,2]. However, increased susceptibility to superficial and invasive *Candida* infections has been reported in these BM-based therapies [1,3]. According to previous studies, long-lasting treatment of asthma bronchiale and chronic pulmonary disease with inhaled glucocorticoids is a potential risk for the development of oral candidiasis [4]. Furthermore, glucocorticoid therapies promote candidiasis in patients who suffer from acute renal failure, cancerous disease, systemic lupus erythematosus or who have undergone bone-marrow or solid-organ transplantation [1,3,5,6]. High dose glucocorticoid treatments with methylprednisolone or BM stimulate hypha formation, as well as the production of extracellular phospholipase and aspartic protease by *Candida albicans* and also stimulate an interaction between *C. albicans* and the gut epithelium resulting in the colonization of the gastrointestinal tract [7,8,9,10]. Hence, new alternative therapeutic approaches are urgently needed to improve the efficiency of anti-*Candida* medication and hereby maintain the quality of life of glucocorticoid-treated patients.

Our previous studies showed glucocorticoid exposures enhance the susceptibility of yeast cells to oxidants such as the superoxide-generating agent menadione sodium bisulfite (MSB; a water-soluble form of menadione) [7,8]. Therefore, the combination of topical glucocorticoid-containing drugs with certain menadione derivatives might be a promising approach for the development of novel antifungal therapeutic strategies, e.g., in dermatology [11,12].

The *C. albicans* protein phosphatase Z1 (CaPpz1) enzyme has been pinpointed as a potential target in healing *Candida* infections of the skin [13]. CaPpz1 has a pivotal role in cation homeostasis, cell wall biosynthesis and the pathogenicity of *C. albicans* [14]. PPZ-type phosphatases are involved in the oxidative stress tolerance of various fungi, including *C. albicans*, *Saccharomyces cerevisiae*, *Aspergillus nidulans* and *Aspergillus fumigatus* [15,16]. Recently we reported that deletion of the fungus specific *CaPPZ1* gene enhanced the sensitivity of *C. albicans* to oxidative stress elicited by *tert*-butyl hydroperoxide (*t*BOOH) [17].

To extend our knowledge of the stress response system of *C. albicans*, the present study has investigated the combined effects of *CaPPZ1* gene deletion and MSB mediated oxidative stress treatments on the physiological and transcriptional responses of the BM-exposed *C. albicans* cultures. Our data suggests that the deletion of the *CaPPZ1* gene and/or the glucocorticoid treatment alone had only slight effects on the transcriptome. In contrast, the combination of MSB treatment and *CaPPZ1* gene deletion resulted in profound global alterations in the gene expression patterns, and the transcriptome changes were even more pronounced when these cells were also treated with glucocorticoid.

## 2. Materials and Methods

### 2.1. Cultivation of Fungal Strains

The control (WT) *C. albicans* QMY23 (his1Δ/his1Δ, leu2Δ:*C. dubliniensis* HIS1/leu2Δ:*C. maltose* LEU2, URA3/ura3Δ:imm^434^, IRO1/iro1Δ:imm^434^) and the phosphatase gene deletion mutant (KO) (ura3Δ-iro1Δ:imm^434^/URA3-IRO1, his1Δ/his1Δ, leu2Δ/leu2Δ, ppz1Δ:*C. dubliniensis* HIS1/ppz1Δ:*C. maltosa* LEU2) strains were constructed earlier by Mitrovich et al. [18] and Ádám et al. [14], respectively. These strains were maintained and cultured on yeast extract-peptone-dextrose (YPD) agar (1% yeast extract (Alfa Aesar, Kandel, Germany), 2% mycological peptone (HiMedia, Mumbai, India), 2% dextrose (VWR International LLC., Debrecen, Hungary) and ±2% agar (VWR International LLC., Hungary), pH 5.6) as described earlier [17]. Unless otherwise indicated, all chemicals were purchased from Sigma-Aldrich Hungary Ltd., Budapest, Hungary.

### 2.2. Stress Treatments

Yeast pre-cultures were grown in 5 mL YPD medium at 37 °C for 18 h, diluted to the optical density of 0.1 at λ = 640 nm (OD_640_) with YPD (defined as 0 h incubation time in growth assays) and the cultures were grown further at 37 °C and at 2.3 Hz shaking frequency.

For testing the long-term physiological responses of BM and/or MSB treated and untreated *C. albicans* strains, selected cultures were also supplemented with 2 mM BM (the water-soluble sodium phosphate derivative) and/or 0.5 mM MSB at 0 h and microbial growth was followed by measuring changes in OD_640_ during a 12 h incubation period [8]. Importantly, the 2 mM BM concentration used in this study was comparable to the glucocorticoid concentrations typically employed in drops, lotions and gels widely used in topical therapies in ophthalmology, dermatology and gynecology.

To study the effects of oxidative stress on short-term transcriptional and physiological responses of the BM exposed and untreated *C. albicans* strains, pre-cultures were diluted to 0.1 OD_640_ in YPD medium, selected cultures were supplemented with 2 mM BM at 0 h and oxidative stress treatments (with or without 1.5 mM MSB) were carried out subsequently, for 1 or 4 h incubation periods. In these experiments, the sublethal MSB concentration of 1.5 mM was chosen because this was the highest oxidant concentration that did not significantly affect the viability of either the WT or the oxidative stress sensitive KO strains within 1 h. After addition of MSB, changes in the OD_640_ and colony forming unit (CFU) values were determined. Samples were taken at 1 h for RNA isolation and for measurement of redox changes and specific enzyme activities, since the response of fungal cells to redox imbalance is a rapidly induced protective mechanism [17]. Four hours MSB stress exposures were used for the determination of the more slowly evolving changes in glucose consumption, ethanol production and metal contents.

### 2.3. RNA Sequencing (RNASeq)

Total RNA samples were isolated from lyophilized *C. albicans* cells (CHRIST Alpha 1−2 LDplus lyophilizer, Osterode, Germany) according to Jakab et al. [19]. Three independent cultures were used for RNASeq experiments and RT-qPCR tests. The quality of RNA was determined using the Eukaryotic Total RNA Nano kit (Agilent, Santa Clara, CA, USA) along with Agilent Bioanalyzer. RNASeq libraries were prepared from total RNA using TruSeq RNA Sample preparation kit (Illumina, San Diego, CA, USA) according to the manufacturer’s protocol. Seventy-five bp long sequence reads were determined on Illumina NextSeq500 instrument. Approximately 16–36 million reads per sample were generated at the Genomic Medicine and Bioinformatics Core Facility of the Department of Biochemistry and Molecular Biology, Faculty of Medicine, University of Debrecen, Hungary. Raw reads were aligned to the reference genome (genome: “http://www.candidagenome.org/download/sequence/C_albicans_SC5314/Assembly22/archive/C_albicans_SC5314_version_A22-s07-m01-r85_chromosomes.fasta.gz”, accessed on 2 July 2021, features: “http://www.candidagenome.org/download/gff/C_albicans_SC5314/archive/C_albicans_SC5314_version_A22-s07-m01-r85_features_with_chromosome_sequences.gff.gz”, accessed on 2 July 2021) and the aligned reads varied between 93–95% in each sample. Tophat (v.2.1.1) and Bowtie (v.2.3.4.1) bioinformatics tools were used for the mapping and generating bam files and htseq-counts (from “HTSeq” framework, version 0.6.1p1) to produce read counts for A and B alleles. The read counts of the corresponding A and B alleles were merged and these values were used for differential expression analysis using DESeq2 (v.1.24.0) according to Love et al. [20]. We used the “DESeq” function to fit a model allowing for batch effects to the data. Only significantly up- and down-regulated genes with a corrected *p* value of <0.05 were considered during the evaluation process. Original data obtained in this work have been deposited in NCBI’s Gene Expression Omnibus [21] and are accessible through GEO Series accession number GSE173668 (https://www.ncbi.nlm.nih.gov/geo/query/acc.cgi?acc=GSE173668, accessed on 5 July 2021).

### 2.4. Reverse Transcription Quantitative Real-Time PCR (RT-qPCR) Assay

Changes in the transcription of selected antioxidant enzyme, membrane transport and primary metabolism genes were validated by RT-qPCR [19]. Oligonucleotide primers (Table S1) were designed with Oligo Explorer (v.1.1.) and Oligo Analyzer (v.1.0.2) software. Relative transcription levels (ΔΔCP value) were calculated as ΔCP_control_ − ΔCP_treated_ where ΔCP_control_ = CP_tested gene, control_ − CP_reference gene, control_ for untreated control while ΔCP_treated_ = CP_tested gene, treated_ − CP_reference gene, treated_ for stress-exposed cultures. The CP values represent the RT-qPCR cycle numbers of crossing points. Three reference genes (*ACT1*, *EFG1* and *RIP1*) were tested. All showed stable transcription in our experiments and, hence, only data relative to the *ACT1* (C1_13700W) transcription values are presented. 

### 2.5. Gene Set Enrichment Analysis

Gene set enrichment analyses were carried out with *Candida* Genome Database Gene Ontology Term Finder (http://www.candidagenome.org/cgi-bin/GO/goTermFinder, accessed on 5 July 2021) using function, process and component gene ontology (GO) terms. Only hits with a *p* value of < 0.05 were evaluated further (Table S2).

Besides GO terms, groups of functionally related genes were also generated by extracting data from the *Candida* Genome Database (http://www.candidagenome.org, accessed on 5 July 2021) unless otherwise indicated. The enrichment of genes from these gene groups were tested with Fisher’s exact test (*p* < 0.05). The following gene groups were created:

(i) antioxidant enzyme genes—this gene group includes genes encoding functionally verified and/or putative antioxidant enzymes including the catalase (GOID: 4096), superoxide dismutase (GOID: 4784), glutaredoxin (GOID: 6749), thioredoxin (GOIDs: 8379 and 51920) and peroxidase (GOID: 4601) GO terms;
(ii) iron metabolism-related genes—genes involved in iron acquisition by *C. albicans* were collected according to Fourie et al. [22];
(iii) zinc and copper homeostasis genes—genes involved in zinc and copper acquisition by *C. albicans* were collected according to Gerwien et al. [23];
(iv) metabolic pathway-related genes—this group contains all genes related to the TCA, ethanol fermentation, glycogen metabolism, and ergosterol biosynthesis biochemical pathways according to the pathway database (http://pathway.candidagenome.org, accessed on 5 July 2021).

The complete gene lists of the above-mentioned gene groups are available in Supplementary Table S3.

### 2.6. Determination of Virulence Attributes

Virulence attributes of BM pre-treated and untreated *C. albicans* cells, including extracellular aspartic proteinase and phospholipase activities, were measured on bovine serum albumin (BSA) and solid egg yolk (EY) medium, respectively, and hypha formation was determined on solid Spider agar by methods described earlier [8,19]. In these assays, pre-cultures were diluted to 0.1 OD_640_ in YPD supplemented with or without 2 mM BM, and the cultures were incubated for 5 h at 37 °C and 2.3 Hz shaking frequency. Fungal cells were collected by centrifugation (5 min, 4000× *g*, 4 °C), washed three times with phosphate-buffered saline (PBS) solution and adjusted to 1 × 10^7^ cells/mL in PBS. The cell suspensions were point-inoculated onto the surface of the selected agar plates and then incubated at 30 °C. Enzyme activities (Pz values) were determined by dividing the colony diameter and precipitation zones on EY plates or clear areas on BSA media-plus-colony diameter after 5 days of incubation at 30 °C. Hypha forming ability on Spider agar was measured after 7 and 10 days incubations, and hyphal growth (%) was calculated using the following formula: (width of hyphal ring)/(colony diameter + hyphal ring) × 100 [8,19].

#### Biofilm Development and Metabolic Activity-Based Susceptibility Testing

One-day-old biofilms were prepared as described previously [24]. Briefly, *C. albicans* cells were suspended in RPMI 1640 liquid medium in a final concentration of 1 × 10^6^ cells/mL and aliquots of 0.1 mL were pipetted onto flat-bottom 96-well sterile microtiter plates (TPP, Trasadingen, Switzerland) and incubated statically at 37 °C for 24 h.

The susceptibility to BM and MSB was tested by using the broth microdilution method according to the CLSI approved standard M27-A3 protocol [25]. The concentration ranges tested for biofilm minimum inhibitory concentration (MIC) determinations were selected between 0.06–4.0 mM and 0.008–2.0 mM for BM and MSB, respectively. The biofilms were washed three times with sterile physiological saline and, MIC determinations were performed in RPMI 1640 using the metabolic activity change-based XTT [2,3-bis(2-methoxy-4-nitro-5-sulfophenyl)-2H-tetrazolium-5-carboxanilide] reduction assay. MIC values were considered as the lowest drug concentrations that caused at least a 50% reduction in metabolic activity compared to untreated biofilms. The interaction between BM and MSB was evaluated using a two-dimensional broth microdilution chequerboard assay against one-day-old biofilms [26]. The nature of the interaction was also analyzed by fractional inhibitory concentration index (FICI) determination. The tested BM and MSB concentrations were the same as those used for the MIC determination. FICIs were calculated by following formula: ΣFIC = FIC_A_ + FIC_B_ = MIC_A_^comb^/MIC_A_^alone^ + MIC_B_^comb^/MIC_B_^alone^, where MIC_A_^alone^ and MIC_B_^alone^ are MICs of compounds A and B when tested alone and MIC_A_^comb^ and MIC_B_^comb^ show the MICs of compounds A and B, respectively, when they are used in combination. FICIs were determined as the lowest ΣFIC values. The interaction between BM and MSB were defined as synergistic when FICI was ≤0.5, as indifferent when FICI was between > 0.5 and 4, and as antagonistic when FICI was >4.

### 2.7. Assays of Redox Homeostasis and Antioxidant Enzyme Activities

Intracellular conversion of 2′,7′-dichlorodihydrofluorescein diacetate (H_2_DCFDA, a non-fluorescent compound) to 2′,7′-dichlorofluorescein (DCF; fluorescent) is a widely used method to determine the redox changes and mitochondrial function [8]. For measuring DCF production and antioxidant enzyme activities, standard unstressed (without or with 2 mM BM) or stressed (1.5 mM MSB added at 4 h culture time) cultures were grown and processed as described below.

For the estimation of DCF production, the fungal cells were supplemented with H_2_DCFDA after 1 h MSB exposure, and samples were taken an hour later, washed with PBS, and re-suspended in 5% sulfosalicylic acid solution. The DCF production was determined spectrofluorimetrically [8].

In order to determine specific antioxidant enzyme activities, *C. albicans* cells harvested after 1 h MSB exposure were thoroughly washed and disrupted using glass beads (1 mm diameter). Glutathione reductase, glutathione peroxidase, catalase and superoxide dismutase activities of the supernatant were determined using the spectrophotometric methods as reported by Jakab et al. [8]. The protein concentration of cell-free extracts was measured by the Bradford method [27].

### 2.8. Assay of Glucose Consumption, Ethanol Production, and Iron, Zinc and Copper Contents of C. albicans Cells

Yeast pre-cultures were grown, and BM pre-treatments and MSB exposures were performed as described above for short-term exposure assays. Aliquots of *C. albicans* culture media were collected by centrifugation (5 min, 4000× *g*, 4 °C) after 4 h incubation following MSB exposure. Fungal dry cell mass (DCM) was measured after freeze-drying the biomass [28].

Changes in the glucose contents of the supernatants were determined by the glucose oxidase assay reported by Leary [29] and Boczonádi et al. [30]. In this reaction rate-based assay the mixtures contained 0.76 mmol/L 4-aminoantipyrine, 11 mmol/L phenol, 4 kU/L glucose oxidase and 1 kU/L peroxidase dissolved in 0.1 mol/L sodium-potassium phosphate (pH 6.6) buffer. The absorbance was measured immediately at λ = 500 nm.

The concentration of ethanol in *C. albicans* culture media was determined by headspace gas chromatography (HS-GC-FID system, PerkinElmer GC, Clarus 680 with PerkinElmer TurboMatrix 40 Trap Headspace Sampler, FID with helium as the carrier gas 1 mL/min) as described previously [31,32]. Static HS injection was made with a 1 µL injection volume, and a capillary column (Agilent, DB−5.625, 30 m × 0.25 mm I.D.) was used for separation. Data analysis was performed with the PerkinElmer TotalChrom Workstation V.6.3.2 Data System.

For element analyses, fungal cells were grown, processed and lyophilized as described above. The metal (Fe, Zn, Cu) contents of the dry biomass were measured by inductively coupled plasma optical emission spectrometry (ICP-OES; 5110 Agilent Technologies, Santa Clara, CA, USA) following atmospheric wet digestion in 5 mL, 65% (M/M) HNO_3_ and 0.5 mL, 30% (M/M) H_2_O_2_ in glass beakers [28,30].

Glucose consumption, ethanol production and metal contents of the samples were calculated and expressed in DCM units (*g*/*g*), as described elsewhere [30].

### 2.9. Statistical Analysis

Unless otherwise indicated, all experiments were performed in triplicates and mean ± standard deviation (SD) values were calculated. Statistical significance of changes was determined by paired Student’s *t* test. Probability levels *p* < 0.05 were regarded as significant. Interaction between the effects of BM and MSB, as well as between MSB and *CaPPZ1* deletion analyzed by two-way ANOVA.

## 3. Results

### 3.1. BM and MSB Suppressed Growth and Metabolic Activity of Biofilm Synergistically

In our long term physiological experiments, the growth rates of the KO strain were always lower than those of the WT in agreement with previous observations [17]. 0.5 mM MSB reduced the growth (OD_640_) of both strains, but MSB had a stronger inhibitory effect on the KO strain (Figure S1). Significant interaction (*p* < 0.05) was also observed between the growth inhibitory effect of BM and MSB, as well as between MSB and *CaPPZ1* deletion for KO cells. Importantly, the combined BM + MSB treatment slowed growth but did not block growth of the deletion mutant completely (Figure S1). This triple perturbation was fungistatic since CFUs were essentially unchanged (in the range of 7.5 ± 0.5 × 10^5^ to 1 ± 0.2 × 10^6^ cell/mL) during the 12 h incubation.

The interaction between BM and MSB was also studied on *C. albicans* biofilms by a two-dimensional broth microdilution chequerboard assay on one-day-old biofilms after 24 h incubation. BM alone had no detectable effect and MSB reduced metabolic activity with a median MIC of 0.25 mM both in the WT and the KO strains. Furthermore, based on calculated median FICI values, a weak synergistic interaction between BM and MSB was observed for the two tested strains (median FICIs were 0.49) (Table 1).

### 3.2. Genome-Wide Transcriptional Changes Confirmed the Interaction between BM and MSB Treatments

**Figure 1:**
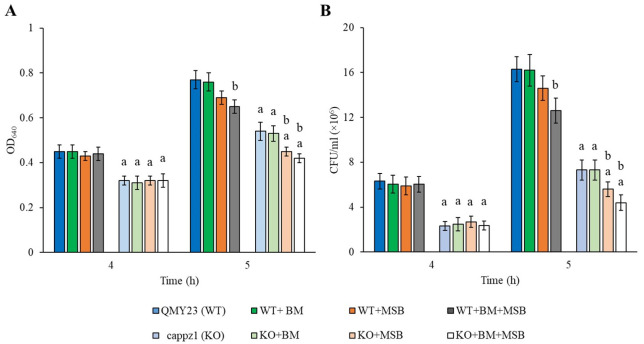
The physiological effect of *C. albicans* protein phosphatase Z1 (*CaPPZ1*) gene deletion, betamethasone (BM) treatment or/and oxidative stress on the growth rate of QMY23 (WT) and cappz1 mutant (KO) *C. albicans* strains. Selected cultures were supplemented with 2 mM BM at 0 h incubation time and oxidative agent (+1.5 mM MSB) at 4 h incubation. The effect of treatments was followed by measuring the optical density (OD_640_) (**A**) and colony forming unit (CFU) (**B**) after 4th and 5th of culturing. Mean ± *SD* values calculated from three independent experiments are presented. ^a^ and ^b^ indicate significant differences at *p* < 0.05 significance level in comparison to WT strain (untreated KO vs. untreated WT and treated KO vs. treated WT) and to untreated controls (treated WT vs. untreated WT and treated KO vs. untreated KO), respectively.

Short term transcriptional responses were studied in eight different cultures: i.e., in untreated control, BM alone, MSB alone, and BM + MSB combined treated WT and KO strains. In these experiments, half of the BM (2 mM) pre-treated and untreated cultures were supplemented with 1.5 mM MSB at 4 h incubation time and samples were collected after 1 h exposure. Figure 1 demonstrates that MSB treatment gave significantly lower OD_640_ and CFU values only in the BM pre-treated WT cultures. In contrast, the same MSB concentration reduced the growth of both BM pre-treated and untreated KO cultures, and additive interaction between BM and MSB as well as between MSB and *CaPPZ1* deletion was observed after 1 h MSB exposure (Figure 1).

**Figure 2:**
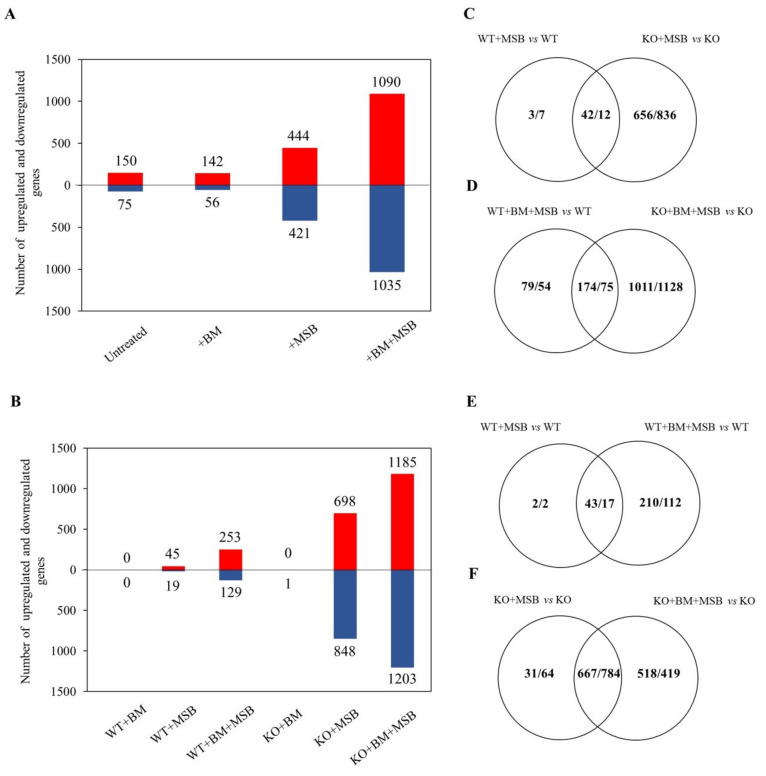
Effects of *C. albicans* protein phosphatase Z1 (*CaPPZ1*) deletion and stress conditions on the transcriptome of *C. albicans*. (**A**) Number of genes up- or down-regulated due to *CaPPZ1* gene deletion alone (untreated) or in the presence of betamethasone (BM), menadione sodium bisulfite (MSB) or BM + MSB as compared to WT under the same experimental conditions. Significantly up- and down-regulated genes with a corrected *p* value of <0.05 were taken into consideration during the evaluation process. (**B**) Number of genes that were up- or down-regulated by BM, MSB or BM + MSB treatments in the QMY23 (WT) or cappz1 mutant (KO) strain. In (**A**,**B**) up- and down-regulated genes are represented by red and blue bars, respectively. (**C**–**F**) Venn diagrams show the number of up-regulated over down-regulated genes under MSB or BM + MSB treatment conditions in four different comparisons. The effects of *CaPPZ1* gene deletion, BM pre-treatment and/or oxidative stress (MSB) on the growth rates are presented in Figure 1.

The mRNA complement of these samples was investigated by RNASeq and RT-qPCR. Reproducible relationships between RNASeq results were confirmed by principal component analysis (Figure S2). The effects of *CaPPZ1* mutation as well as those of BM and MSB exposures on the transcriptomes are summarized in Figure 2. Deletion of *CaPPZ1* induced a moderate change in the transcriptome even in untreated or BM pre-treated cultures and the difference between the KO and WT strains increased substantially after MSB treatments (Figure 2A). Significant overlaps were found between the two strains but the oxidative stress altered the expression of additional genes in the KO (Figure 2C,D). BM pre-treatment alone hardly affected the gene expression in WT or KO strains (Figure 2A,B). The effect of MSB treatment was more pronounced on the *CaPPZ1* deleted strain because the expression of 24 times more genes was altered in the KO mutant strain than in WT reference culture (45/19 and 689/848 genes up/down in the control and the mutant strains). Importantly, BM pre-treatment markedly increased the number of differentially expressed genes in both the MSB treated WT and KO cells (253/129 vs. 1185/1203 genes up/down in WT vs. KO) (Figure 2B). The overlaps between MSB and BM + MSB responsive genes were considerable (43/17 vs. 667/784 genes up/down in WT vs. KO) (Figure 2E,F), but the expression of several genes changed only in response to combined BM + MSB treatments.

A significant portion of the RNASeq-based gene expression data were confirmed by RT-qPCR analyses. The transcriptional changes of 16 genes selected for RT-qPCR testing showed a strong positive correlation between the results obtained for both methods (Table S4). Pearson’s correlation coefficients were in the range of 0.73–0.90 for both strains.

### 3.3. Gene Ontology Analysis of the Transcriptome Changes

**Figure 3:**
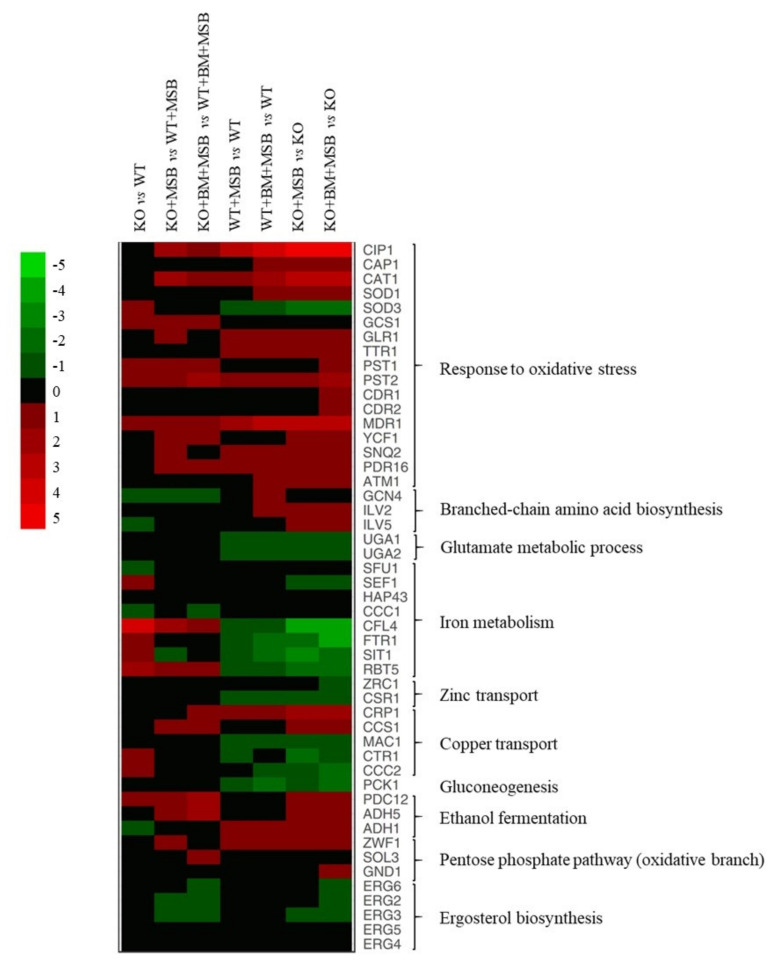
The effects of *C. albicans* protein phosphatase Z1 (*CaPPZ1)* deletion and stress conditions on the expression of selected genes of *C. albicans*. The heat map demonstrates the expression profiles of representative genes according to the color scale that indicates gene expression changes in log_2_FC units. Supplementary Table S3 summarizes the data that were used for the construction of the heat map.

The possible physiological background of the transcriptional changes for upregulated and downregulated genes was further characterized using gene set enrichment analyses (Table 2, Tables S2 and S3) and selected changes are illustrated in a heat map (Figure 3).

#### 3.3.1. The CaPPZ1 Gene Deletion Has a Moderate Transcriptomic Effect

The lack of CaPpz1 protein phosphatase alone resulted in up-regulation of 11 iron homeostasis-related genes (e.g., *CFL4*, ferric reductase *SIT1*, siderophore transporter, and *RBT5*, hemoglobin utilization) and 27 genes encoding oxidoreductases (e.g., *PST1* and *PST2*, flavodoxin-like proteins, *SOD3*, superoxide-dismutase, and *GCS1*, γ-glutamyl cysteine synthase) under standard unstressed culture conditions (Figure 3, Table 2, Tables S2 and S3). The upregulation of *CFL4* and *SOD3* was supported by RT-qPCR data (Figure 3 and Table S4).

Deletion of the *CaPPZ1* gene down-regulated seven genes of glycolysis, five genes of gluconeogenesis, as well as nine genes of alpha-amino acid metabolism including four genes of branched-chain amino acid synthesis (e.g., *GCN4*, transcription factor) (Figure 3 and Table 2).

#### 3.3.2. The Cellular Response to the Oxidative Stress Was More Extensive in the Cappz1 Knock Out Strain

Altogether, 12 oxidative stress response genes were up-regulated in the WT strain after exposure to MSB (e.g., genes of *CIP1* oxidoreductase, *TRR1* thioredoxin reductase, *MDR1* and *YCF1* ABC transporters) (Figure 3, Table 2, Tables S2 and S3). Upregulation of *MDR1* and *TRR1* under MSB stress in control and BM pre-treated strains was also confirmed by RT-qPCR data (Table S4). MSB treatment down-regulated glutamate metabolism (*UGA1*, GABA transaminase, and *UGA2*, succinate semialdehyde dehydrogenase), and *PCK1* encoding a key enzyme specific to gluconeogenesis in the WT strain (Figure 3, Table 2, Tables S2 and S3). Downregulation of *PCK1* under MSB and BM + MSB treatments in both strains was confirmed by RT-qPCR data (Table S4).

In the case of the KO mutant, 43 genes related to oxidative stress response, nine genes of branched-chain amino acid synthesis (e.g., *GCN4*, transcription factor, *ILV2* and *ILV5*, isoleucine biosynthesis enzymes), copper access (*CRP1*, transporter, and *CCS1*, chaperone), glycolysis (14 genes), ethanol fermentation (four genes), glycogen biosynthesis (four genes), and 23 genes of peroxisome proteins (including peroxisome related and fatty acid β-oxidation genes) were significantly enriched in the up-regulated gene set (Figure 3, Table 2, Tables S2 and S3). Genes involved in iron homeostasis (19 genes), copper uptake (*MAC1*, transcription factor; *CTR1* and *CCC2*, transporters), zinc metabolism (*CSR1*, transcription factor, and *ZRC1*, transporter) as well as in RNA metabolism (252 genes), the ribosome (73 genes), RNA transport (37 genes) and the mitochondrion (145 genes) were enriched in the down-regulated gene set (Figure 3, Table 2, Tables S2 and S3). The KO + MSB vs. WT + MSB comparison indicated that oxidative stress decreased the expression of eight ergosterol biosynthesis pathway related genes in KO cells (Table 2).

Upregulation of three oxidative stress response genes (*CAT1*, catalase; *SOD1*, cytosolic Cu and Zn-containing superoxide dismutase, and *GLR1*, glutathione reductase) and downregulation of *SOD3* (coding for cytosolic Mn-containing superoxide dismutase), *CFL4* (ferric reductase) and *FTR1* (iron permease) iron homeostasis-related genes in untreated and BM pre-treated KO cells were also confirmed by RT-qPCR (Figure 3 and Table S4).

#### 3.3.3. Combination of CaPPZ1 Gene Deletion and MSB Stress Led to a Global Reprogramming of Gene Expression in BM Pre-Treated Cells

MSB treatment resulted in enrichment of the up-regulated oxidative stress response (altogether 26 genes e.g., *SNQ2* and *PDR16* encoding ABC transporters) and alpha-amino acid metabolic process (21 genes) genes and, concomitantly resulted in the downregulation of transmembrane transport (28 genes) including H(+)-ATPase (*PMA1*), carbohydrate transport (6 genes), and iron transport (9 genes), as well as two key genes specific to gluconeogenesis (like *PCK1*) in the BM pre-treated WT cultures (Figure 3, Table 2, Tables S2 and S3).

The same treatment significantly enriched the up-regulated oxidative stress response (54 genes e.g., *CDR1*, *CDR2*, and *MDR1*), peroxisome (35 genes), alpha-amino acid synthesis (51 genes), ethanol fermentation (7 genes), and fatty acid catabolism (12 genes) in the KO strain. At the same time, RNA metabolism (325 genes), the ribosome (95 genes), RNA transport (51 genes), and the mitochondrion (201 genes) were enriched in the downregulated gene set (Figure 3, Table 2, Tables S2 and S3). The mutation also caused a significant increase in the expression of *CDR1* (ABC transporter), *PXP2* (acyl-CoA oxidase enzyme of fatty acid β-oxidation) and *HGT17* (hexose transmembrane transporter) of BM + MSB treated cells according to the RT-qPCR measurements (Table S4). In addition, the combined treatment increased the expression of oxidative branch of the pentose phosphate pathway-related genes (*ZWF1*, *SOL3*, and *GND1*) (Figure 3).

### 3.4. Testing of the Predicted Consequences of Transcriptome Changes

Some of the possible physiological consequences deduced from the transcriptomic changes described in the previous sections were scrutinized in the following experiments.

#### 3.4.1. BM Did Not Influence the Growth, Extracellular Phospholipase and Proteinase Production and Hypha Formation of *C. albicans*

As BM did not affect the gene expression of *C. albicans*, first we studied the physiological effects of BM on the wild-type and deletion mutant strains after 5 h steroid exposure. We found that the BM pre-treatment did not affect the growth (OD_640_) of *C. albicans* cells at the pharmacologically relevant concentration of 2 mM (*p* > 0.05) (Figure 1). Furthermore, it did not significantly influence the extracellular proteinase and phospholipase activities and the hypha formation of the tested *C. albicans* strains (*p* > 0.05). On the other hand, as expected, deletion of *CaPPZ1* significantly reduced the yeast to hyphae morphological transitions compared to the reference strain (Table 3).

#### 3.4.2. BM Pre-Treatment Enhanced the Oxidative Stress and the Stress Response Induced by MSB

The BM, MSB and BM + MSB treated cultures were characterized by changes in the activities of antioxidant enzymes and redox homeostasis (DCF production). The deletion of *CaPPZ1* facilitated the formation of DCF and increased the specific glutathione reductase and glutathione peroxidase activities in comparison to untreated WT cultures, in line with previous observations [17]. In contrast to BM exposures, MSB treatments led to the development of oxidative imbalance and significantly increased the specific activities of the tested antioxidant enzymes in both WT and KO strains (Table 4). It is important to note that combined BM + MSB treatments had significantly stronger effects than MSB exposures alone on DCF production with the WT and KO strains, and on enzyme activities of the KO strain (Table 4). Furthermore, significant interaction (*p* < 0.05) was also observed between BM and MSB as well as MSB and *CaPPZ1* deletion on the DCF production of both strains, as well as between BM and MSB on the glutathione reductase, glutathione peroxidase and catalase activities of KO cells (Table 4).

#### 3.4.3. Deletion of CaPPZ1 and MSB-Induced Oxidative Stress Reduced Glucose Consumption, Ethanol Production and Influenced Metal Ion Content of *C. albicans*

**Figure 4:**
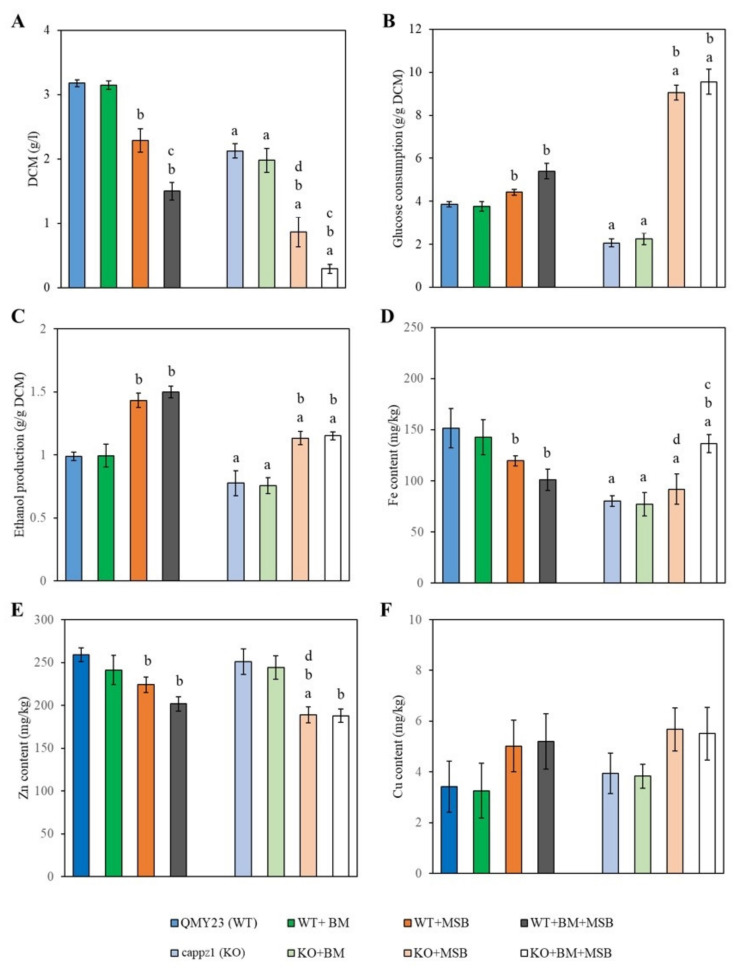
The physiological effects of *C. albicans* protein phosphatase Z1 (*CaPPZ1*) gene deletion, betamethasone (BM) pre-treatment and/or menadione sodium bisulfite (MSB) on the growth (**A**), glucose utilization (**B**), ethanol production (**C**) and metal content (**D**–**F**) of the QMY23 (WT) or cappz1 mutant (KO) *C. albicans* strains. Cell cultures were supplemented with 2 mM BM at 0 h incubation time and oxidative stress (+1.5 mM MSB) was initiated at 4 h incubation time. Samples were taken after 4 h of MSB exposure. Data represent mean values ± SD calculated from 3 independent experiments. ^a^ Significant difference between untreated KO vs. untreated WT and treated KO vs. treated WT. ^b^ Significant difference between treated WT vs. untreated WT and treated KO vs. untreated KO. ^c^ Significant interaction between the effects of BM and MSB according to two-way ANOVA in the studied strain. ^d^ Significant interaction between the effects of MSB and the deletion of *CaPPZ1* according to two-way ANOVA (BM pre-treated cultures were omitted from the calculations).

Glucose consumption, ethanol fermentation, as well as intracellular metal contents of *C. albicans* were analyzed after 4 h MSB exposure (Figure 4) because neither glucose consumption nor ethanol fermentation changed significantly after 1 h MSB exposure. It is worth noting that BM alone had no significant effect on either the strains or the physiological parameters tested. Significant decrease was detected in the growth (DCM), ethanol fermentation and the iron content of the untreated and oxidative stress treated KO cells when compared to the appropriate WT cultures (Figure 4A,C,D).

MSB treatment decreased the growth and zinc content of the KO strain plus the iron contents of WT cells (Figure 4A,D,E). Glucose utilization and ethanol production increased in both MSB-exposed WT and KO strains (Figure 4B,C). Combined BM + MSB treatments had a more significant impact on the growth of both strains (Figure 4A) and the iron content of the KO cells than MSB exposures alone (Figure 4D). The copper content of *C. albicans* cells measured in the presence of BM, MSB and BM + MSB did not differ significantly from the untreated control cultures (Figure 4F).

## 4. Discussion

Deeper insight into the physiological and molecular mechanisms of new antifungal candidates may help to develop safe and effective antimycotics to combat infections caused by *C. albicans*. Use of global transcriptome analysis has provided a platform to compare and functionally characterize the effects of *CaPPZ1* gene deletion and concomitant oxidative stress exposure on BM pre-treated *C. albicans.* Our previous studies indicated that the BM concentration used in this study (2 mM) was not toxic for oral, intestinal and vaginal epithelial cell lines [9]. In addition, we observed that 2 mM BM pre-treatment of the fungal cells did not significantly impact on either physiology or virulence and had only minor effects on the interaction of *C. albicans* with human epithelial cells [8,9]. In accordance with these observations, we did not find any significant differences in the physiological and global transcriptional responses of BM treated versus untreated *C. albicans* strains. However, BM sensitized the fungal cells’ response to the oxidizing agent MSB as we reported earlier [7,8] and extended in the present work. Here we report that the negative effect of the fungus specific protein phosphatase *CaPPZ1* deletion on the oxidative stress tolerance of *C. albicans* interacts significantly with BM exposure.

### 4.1. Phenotypes and Transcriptional Changes Attributed to CaPPZ1 Gene Deletion

The involvement of *CaPPZ1* in oxidative stress response has been studied extensively and reported earlier [15,17]. Our study of the transcriptome changes in the KO indicated indeed some alteration in gene expression related to the oxidative stress response (Table S3), including flavodoxin-like proteins, superoxide-dismutase and γ-glutamyl cysteine synthase. These proteins confer important antioxidant effects that are critical for fungal virulence and are needed to combat oxidative stress [33,34]. Upregulation of these genes as well as the elevated DCF and antioxidant enzyme activities (Table 4), in agreement with Szabó et al. [17], also suggest that the phosphatase deletion and oxidative stress tolerance were tightly linked in *C. albicans*, similarly to that found in *S. cerevisiae*, *A. fumigatus* and *A. nidulans* [15,16]. The upregulation of several genes related to “redox processes” and “response to oxidative stress” upon the overexpression of *PPZ1* in *S. cerevisiae* [35], providing further support for the intimate correlation between oxidative stress and the Ppz enzymes. Moreover, mitochondria are the major site of reactive oxygen species (ROS) production. According to several publications the ROS generation in yeast cells contributes to mitochondrial damage [36,37,38,39,40]. Our data support the assumption that the deletion of *CaPPZ1* may led to mitochondrial dysfunction.

In addition, two previous studies pointed to the possible role of Ser/Thr phosphatase Z in the iron metabolism of the opportunistic human pathogenic fungi *A. fumigatus* and *C. albicans* [17,41]. Furthermore, the *A. fumigatus* Δ*ppzA* mutant exhibited reduced growth under iron starvation conditions but not under untreated or iron excess conditions. These genes related to reductive iron uptake and siderophore biosynthesis were up-regulated after iron starvation [41,42]. Nevertheless, the loss of *C. albicans CaPPZ1* resulted in a significant overall decrease in the iron content of KO cells (Figure 4D) and, as a consequence, *CaPPZ1* deletion elevated the expression of several iron homeostasis-related genes (Table 2 and Table S3). These included essential elements of reductive iron uptake (*CFL2*, *CFL4*, *CFL5*, *FRP1*, *FRE10*, *FTH1*, *FET31*, and *FET99*), siderophore transport (*SIT1*), and hemoglobin utilization (*RBT5*, and *PGA7*). We noted that *CaPPZ1* deletion did not affect the expression of *SEF1* and *HAP43* (Figure 3, Table S3) which are essential for low iron-responsive transcriptional regulation in *C. albicans* [43]. Consequently, CaPpz1 seems to be a key player in the maintenance of the iron homeostasis of *C. albicans*. Although its deletion caused reduced iron accumulation within the fungal cells that led to the general upregulation of the iron acquisition pathways, the target proteins of CaPpz1 modulating the iron metabolism are yet to be elucidated.

The upregulation of several plasma membrane-associated genes in the KO strain is in line with previous publications reporting on the role of Ppz1 phosphatase in transmembrane transport processes of *C. albicans* [17] and in other fungi [44]. This effect becomes apparent when the two strains are compared under stress conditions, i.e., in the presence of MSB, and even more significantly in combined BM plus MSB treatment (Table 2).

It is important to note that significant inhibitory effects on the growth and hypha formation of *C. albicans* (Figure 1 and Table 3) were also recorded in the KO strain, which was in good accordance with our previous results [14,17,45].

### 4.2. Transcriptional Changes Related to MSB Exposures

Based on our RNASeq data, MSB stress elicited only a moderate effect on the transcriptome of WT cells as the expression of only 64 genes changed significantly (45/19 genes up/down) (Figure 2, Table S3). In comparison, the transcriptomic effect of mild *t*BOOH (0.4 mM) treatment was more pronounced (132/64 genes up/down-regulated more than two-fold), including the upregulation of translation, RNA metabolism, and downregulation of cell surface, oxidoreductase activity associated genes [17]. Chen et al. [46] demonstrated that the transcriptomic response of fission yeast to MSB was also less pronounced than *t*BOOH or H_2_O_2_, as indicated by the five times higher number of differentially expressed genes counted under *t*BOOH (2 mM) or medium H_2_O_2_ dose (0.5 mM) stress.

In our study, the differentially expressed genes were mainly involved in the antioxidative defense of *C. albicans* (Table 2). The *MDR1* gene coding for a multidrug ABC transporter and the *CIP1* gene encoding an environmental stress induced protein [47] were significantly up-regulated by MSB (Figure 3, Table S3) and, most likely, contributed to the protection of *C. albicans* cells against oxidative stress. In addition, our data support the idea that MSB may be a potential substrate for the Mdr1 efflux pump [47,48]. Upregulation of genes encoding antioxidative enzymes is a predictable response to oxidative stress, i.e., increased specific activities of catalase, glutathione peroxidase, and glutathione reductase (Table 2, Table 4 and Table S3) in agreement with previous publications [17,19,49,50,51].

In addition, our data demonstrated that MSB stress also affected the transcription of iron homeostasis-related genes (Figure 3 and Table 2), as well as the iron and zinc content of the WT strain (Figure 4). The downregulation of iron uptake genes was likely associated with the significantly decreased iron contents measured in MSB-exposed cells. Importantly, this response may constitute part of the protective oxidative stress response that minimized the oxidative damage caused by the ferrous ions. It is well-known that elevated free intracellular iron levels facilitate the formation of ROS and mediate iron-dependent cell death in baker’s yeast [52]. Meanwhile, the down-regulated expression of *CSR1*, encoding a major transcription factor that stabilizes zinc homeostasis and provides cells with zinc-dependent protection against oxidative stress [23], is probably consistent with the observed decreased intracellular zinc levels (Figure 3 and Figure 4).

### 4.3. Combined Effects of CaPPZ1 Deletion and MSB Treatment

As expected, the KO strain showed elevated oxidative stress sensitivity in flask cultures in the presence of MSB in comparison to the genetically matched QMY23 WT strain (Figure 1 and Table 4).

The *CaPPZ1* deletion significantly enhanced the number of up-regulated oxidative stress response genes (Table 2, Tables S2 and S3), which correlated with increased antioxidant enzyme activities (Table 4) [17]. The KO + MSB vs. WT + MSB comparison also demonstrated that *CaPPZ1* deletion up-regulated the transmembrane transport genes and decreased the expression of several ergosterol metabolism related genes under MSB exposure (Table 2).

The general downregulation of RNA metabolism and/or ribosome biogenesis is a common element of the oxidative stress responses of various fungi [19,49,50,53] that is readily detectable in the more sensitive mutant background (Table 2 and Table S2) in agreement with Szabó et al. [17]. MSB-exposed cells can save energy and nutrients through the downregulation of these processes that prevent or ameliorate oxidative injury of macromolecules in *C. albicans* cells [54].

It is noteworthy that *CAP1* coding for one of the major regulators in oxidative stress defense, as well as *ATM1*, *YCF1/MLT1*, *SNQ2* ABC transporters, *MRD1* multidrug efflux pump and *PDR16* phosphatidylinositol transfer protein [51,55], were induced after exposing the KO strain to MSB (Figure 3, Table S3). The ABC transporters and their transcriptional regulators are known to be essential for adaptation to environmental changes [55]. The *SNQ2* and *YCF1* transporter genes are among the primary targets of the Yap1 homolog Cap1 bZIP-type transcription factor and are rapidly expressed in response to oxidative stress [55]. Ycf1 is a vacuolar ABC transporter, which probably transports glutathione S-conjugates into the vacuoles [55,56]. Moreover, the phosphatidylinositol transfer protein Pdr16, may provide *C. albicans* cells with protection against MSB via sensing of stress-dependent changes in sterol biosynthesis and membrane lipid composition, and modulating the activity of enzymes involved in ergosterol biosynthesis [57].

Oxidative stress also caused profound alterations in the primary metabolism of the KO strain (Table 2 and Table S3). For instance, the transcription of several genes related to glycolysis, glycogen and amino acid metabolism was up-regulated (Table 2, Tables S2 and S3). Such metabolic pathways are known to contribute to the stress defense of *C. albicans* [58,59].

Moreover, upregulation of peroxisome-related process was also observed under MSB stress (Table 2 and Table S2), as shown before for *t*BOOH treatments [17]. This was indicative of the progressive degradation of damaged, oxidative stress sensitive unsaturated fatty acids [51,56].

MSB exposure influenced the regulatory pattern of several genes related to copper and zinc metabolisms (Figure 3, Table S3) and affected the metal content of KO cells (Figure 4). Downregulation of a set of genes related to Cu^2+^-influx (*CTR1*, *CCC2* and *MAC1*), and the concomitant upregulation of *CRP1*, *CCS1*, and *SOD1* were likely to have been elicited by Cu^2+^ influx under MSB exposure (Figure 3, Table S3) [60,61,62,63,64,65]. Although the Cu contents of MSB-exposed cultures were somewhat higher than those of their unstressed counterparts, these differences were not statistically significant and clearly indicated that the efficiency of the copper exclusion system under oxidative stress (Figure 4F). Copper plays a major role in innate immune functions against prokaryotic and eukaryotic microbial pathogens [23]. Previous infection studies suggested that macrophages elicit oxidative stress in the pathogens and, concomitantly, also accumulated Cu^2+^ ions inside microbe-containing phagosomes via the upregulation of Ctr1 and the P-type copper ATPase ATP7A [66,67,68]. Recent studies of *C. albicans* demonstrated that metallothioneins, copper efflux pumps and ROS detoxification mechanisms may be equally important in fungal defense strategies against phagocytes [61,65,67,68]. Similar to the alterations in the zinc homeostasis of the WT strain, MSB treatment also decreased the zinc content of the KO cells (Figure 4E) and downregulated the *CSR1* zinc homeostasis transcription factor (Figure 3, Table S3). Hence, the observed oxidative stress sensitivity of the KO strain can be attributed, at least in part, to the observed low zinc levels [23].

### 4.4. Synergistic Effects of BM Exposure and MSB Treatment on the Phosphatase Deletion Mutant

Our growth and biofilm-based experiments detected synergy between MSB and BM exposures in the *C. albicans* cultures tested (Figure S1 and Table 1), in line with our previous findings with the *C. albicans* SC5314 strain [8]. In case of biofilms, a weak synergistic interaction was observed based on median FICI value derived from three independent experiments (Table 1). It should be pointed out that one of the problems of assessing antifungal combinations by the FICI is the choice of the endpoint. To date, there is no solid consensus about which endpoint should be used, particularly when one of the drugs tested is not a traditional antifungal agent, which is the case in this study. In addition, it is particularly true in the case of biofilms where metabolic activity-based susceptibility tests are generally used [69]. Based on the molecular background of the synergism, BM pre-treatment of *C. albicans* cells significantly influenced and enhanced the gene expression changes recorded in MSB-treated WT cells, whereas MSB alone showed moderate effects on the transcripts’ levels (Figure 2 and Figure 3, Table 2). Similarly, the combined effects of *CaPPZ1* gene deletion and MSB-elicited stress led to a global retailoring of the gene expression pattern in BM pre-treated KO mutant (Figure 2 and Table 2), consistent with the observed remarkable growth inhibition (Figure 1 and Figure S1).

Importantly, a wide spectrum of disadvantageous physiological changes taking place in high-dose (4 mM) methylprednisolone sodium hemi-succinate exposed *C. albicans* cells has been reported by Gyetvai et al. [7], and these changes included significantly decreased membrane fluidity. Menadione, redox cycling and arylating quinone [49,70], is also considered as a membrane-active compound, that modulates membrane fluidity in a concentration-dependent manner, e.g., membrane fluidity has been reported to increase at high menadione concentrations [71]. On the other hand, menadione can lower membrane fluidity at low concentrations [72] and reduce membrane integrity via depletion of intracellular thiol pools [73].

The downregulation of ergosterol biosynthesis genes was more likely to have altered the membrane permeability and fluidity. In our study, the *ERG6* gene was only down-regulated following MSB exposure of the BM pre-treated KO cells (Figure 3, Table 2). The reduction in *ERG6* was likely to have increased the passive diffusion of BM across the membrane [74,75]. In addition, the downregulation of *ERG6* may confirm the increased susceptibility to oxidative stress of yeast [74,75,76,77].

The *PMA1* gene was also down-regulated in the response to BM + MSB. It is well-established that Pma1 H(+)-ATPase contributes to several critical physiological functions by maintaining intracellular pH and plasma membrane potential of yeast cells [78,79,80]. We suggest that the downregulation of *PMA1* affected nutrient uptake which correlates with the inhibition of cell growth.

Concerning iron uptake, a significant synergistic effect between BM and MSB exposures was observed in KO cultures (Figure 4D). The elevated free iron levels in the KO cells were most likely to have contributed to the increased redox imbalance and mitochondrial dysfunction quantified by DCF production (Table 4), which was accompanied by increases in the specific activities of glutathione reductase, glutathione peroxidase, catalase, and superoxide dismutase similar to that found in MSB treated KO cultures (Table 4).

It is reasonable to assume that the synergism observed in the anti-*Candida* effects of glucocorticoids and MSB can be attributed to their complex, mutually reinforcing effects on membrane fluidity and integrity but this hypothesis needs further testing.

Under BM + MSB stress conditions, the KO strain used the several stress response processes to mitigate the effects of the combined stress exposure. *C. albicans* cells utilized fatty acids degraded via β-oxidation. The elimination of the unnecessary or damaged membrane lipids and the increased utilization of fatty acids may ensure a higher metabolic flux needed for survival such as the maintenance of membrane fluidity and growth of pathogenic microorganism [81]. In addition, the combined stresses significantly enriched the number of up-regulated oxidative stress response genes including *CDR1* and *CDR2* in KO cells (Figure 3, Table 2 and Table S3). The drug efflux ABC transporters have similar functions to those of *S. cerevisiae* Pdr5, one of the primary drug efflux pumps operated in response to oxidative stress, and it may participate in the transport of either BM or MSB/ROS-modified metabolites and MSB out of the cells [55,82]. Furthermore, in the glucose shunt pathway, *ZWF1* and *GND1* genes encoding glucose−6-phosphate 1-dehydrogenase and 6-phosphogluconate dehydrogenase, respectively, were up-regulated to ensure the continuous supplementation of *C. albicans* with NADP(H), which is essential for the NADP(H) cofactor-dependent oxidative stress responsive enzymes in the glutathione, glutaredoxin and thioredoxin systems [83,84].

## 5. Conclusions

The widespread application of corticosteroids including BM sets the stage for an effective medical intervention that unwittingly predisposes patients for potentially hazardous *Candida* colonization [1,9]. According to our results the steroid has no detectable effects on the pathogen, but makes it more sensitive to oxidative stress generated by MSB [8,9]. Our current experimental data provide important insight into the mechanism of MSB action and also shed light on the molecular background of the synergistic inhibition of *C. albicans* by BM and MSB. Previously we reported that low doses of BM did not have significant damaging side effects on three human cell lines [9], and here we confirmed that the steroid alone had no measurable effects on the transcriptome, biochemistry and physiology of the fungal pathogen. We also revealed some of the possible mechanisms that underline the positive interaction between BM and MSB. However, the apparent lack of target specificity of the oxidative treatment might set limits to its medical application. In a strategy designed to avoid the steadily evolving resistance against presently used antifungals, we proposed that the required specificity of intervention could be assured by targeting the fungus specific CaPpz1 phosphatase involved in the virulence and oxidative stress response of several pathogenic fungi [13]. In our previous studies [14,17] and current experiments (Figure 2 and Table 2), we noted that the deletion of the *CaPPZ1* gene exerted only a moderate effect on the physiology and gene expression profile of *C. albicans*. We also demonstrated that the lack of CaPpz1 triggered more robust changes in the BM pre-treated fungal cells. Even more importantly, we found that the combination of BM with the MSB-induced oxidative stress and the phosphatase deletion together elicited global gene expression changes in the opportunistic pathogen and had a strong fungistatic effect (Figure 2 and Table 2). In conclusion, our findings suggest that the interaction between the inactivation of the fungus-specific phosphatase and the oxidative treatment together can be exploited in the development of a novel topical anti-*Candida* therapy for BM treated patients. The main caveat of this approach is that *PPZ* specific inhibitors are not yet available. However, determination of the three-dimensional structure of the C-terminal catalytic domain [85], and site-directed mutagenesis based mapping of the N-terminal regulatory domain [86] of CaPpz1 pinpoint unique elements in the protein structure. These findings offer a possibility of designing precision inhibitors, which would not interfere with the conserved human protein phosphatase 1 catalytic subunit, and thus could avoid unwanted side effects stemming from the inhibition of an essential host phosphatase.

## Figures and Tables

**Table 1:** Betamethasone (BM) or/and menadione sodium bisulfite (MSB) susceptibility of *C. albicans* biofilms.

Strains	Median (Range) MIC Values				Interaction	
	MIC Alone		MIC in Combination		Median (Range) FICI	Type of Interaction
	BM (mM)	MSB (mM)	BM (mM)	MSB (mM)		
QMY23 (WT)	>4 ^a^	0.25	2 (2–4)	0.06 (0.03–0.06)	0.49 (0.49–1)	Synergy
cappz1 (KO)	>4 ^a^	0.25	0.125 (0.125–0.5)	0.06 (0.008–0.06)	0.49 (0.302–0.49)	Synergy

Median (range) values calculated from three independent experiments are presented. ^a^ Minimum inhibitory concentration (MIC) is off-scale at >4 mM, 8 mM (one dilution higher than the highest tested concentration) was used for fractional inhibitory concentration index (FICI) analysis.

**Table 2:** Summary of gene enrichment analyses.

Significantly Enriched Gene Groups (Number of Genes Associated with the Group)	Number of Up- and Down-Regulated Genes (with Corrected *p*-Value) ^a^						
	KO vs. WT	KO + MSB vs. WT + MSB	KO + BM + MSB vs. WT + BM + MSB	WT + MSB vs. WT	WT + BM + MSB vs. WT	KO + MSB vs. KO	KO + BM + MSB vs. KO
Transmembrane transport ^b^ (365)		**46**(3 × 10^−2^)	**103**(3 × 10^−5^)		**27**(9.9 × 10^−7^)		
Plasma membrane ^b^ (509)	**32** (1 × 10^−5^)		**132** (1.3 × 10^−5^)		**28** (6.6 × 10^−5^)		
Carbohydrate transport ^b^ (34)		**11**(9 × 10^−3^)	**20**(7.4 × 10^−5^)		**6**(2.6 × 10^−2^)		
Oxidoreductase activity ^b^ (429)	**27** (2 × 10^−4^)	**64**(2.9 × 10^−7^)	**127** (3.9 × 10^−9^)	**21** (5.3 × 10^−12^) **8** (5 × 10^−3^)	**60**(7.24 × 10^−17^)	**110** (5.6 × 10^−17^)	**165**(9.7 × 10^−22^)
Response to oxidative stress ^b^ (153)		**27**(4 × 10^−3^)		**12** (7.2 × 10^−8^)	**26**(1.1 × 10^−7^)	**43** (2.5 × 10^−6^)	**54** (1 × 10^−3^)
Antioxidant activity ^c^ (58)		**13**(1.1 × 10^−4^)	**17** (1.3 × 10^−2^)	**6**(2.5 × 10^−6^)	**16** (3.4 × 10^−10^)	**23** (8.9 × 10^−9^)	**26**(2.8 × 10^−6^)
Peroxisome ^b^ (70)		**18**(1 × 10^−4^)	**38** (2.6 × 10^−10^)			**23** (2.0 × 10^−4^)	**35** (5.0 × 10^−7^)
RNA metabolic process ^b^ (776)		**92** (2.5 × 10^−6^)	**231** (5.4 × 10^−22^)			**252** (7 × 10^−49^)	**325** (4.1 × 10^−56^)
Ribosome ^b^ (194)		**76** (4.6 × 10^−39^)	**131** (7.8 × 10^−58^)			**73**(4.7 × 10^−16^)	**95**(6.3 × 10^−20^)
RNA transport ^b^ (114)			**47** (1.4 × 10^−7^)			**37** (8.4 × 10^−5^)	**51** (1.6 × 10^−7^)
Alpha-amino acid metabolism ^b^ (132)	**9** (8 × 10^−3^)				**21**(2.3 × 10^−5^)	**41**(2.1 × 10^−7^)	**51** (5.2 × 10^−5^)
Branched-chain amino acid biosynthesis ^b^ (12)	**4** (3 × 10^−3^)				**6**(2 × 10^−3^)	**9**(1 × 10^−3^)	**10**(4 × 10^−3^)
Glutamate metabolic process ^b^ (11)				**2**(3 × 10^−2^)			
Mitochondrion ^b^ (649)						**145** (1.5 × 10^−9^)	**201** (2.2 × 10^−13^)
Iron homeostasis-related genes ^c^ (48)	**11** (7.8 × 10^−9^)	**7**(4 × 10^−2^)		**5**(2 × 10^−7^)	**9** (2.4 × 10^−8^)	**19**(8 × 10^−7^)	**18**(4 × 10^−3^)
Glycolytic process ^b^ (17)	**7**(1.6 × 10^−7^)					**14** (2.1 × 10^−8^)	**16** (4.4 × 10^−8^)
Gluconeogenesis ^b^ (9)	**5** (9.3 × 10^−6^)						
Ethanol fermentation pathway ^c^ (8)			**4** (3 × 10^−2^)			**4**(7 × 10^−3^)	**7** (4.6 × 10^−5^)
Glycogen biosynthesis pathway ^c^ (7)			**6**(2.6 × 10^−5^)			**4**(7 × 10^−3^)	**5**(7 × 10^−3^)
Fatty acid catabolic process ^b^ (18)			**15** (2.3 × 10^−6^)				**12**(2 × 10^−2^)
Ergosterol biosynthesis pathway ^c^ (20)		**8** (1.9 × 10^−5^)	**7** (3 × 10^−2^)				

^a^ Bold numbers represent the up-regulated (red) or down-regulated (blue) genes belonging to gene groups in comparisons where the enrichment was significant; *p* values are given in parentheses. Up- and down-regulated genes were defined as differentially expressed genes (corrected *p* value of <0.05). ^b^ Selected significant Gene Ontology (GO) terms (*p* < 0.05) were identified with the aid of *Candida* Genome Database Gene Ontology Term Finder (http://www.candidagenome.org/cgi-bin/GO/goTermFinder, accessed on 5 July 2021). The full list of the significantly enriched GO terms is available in Supplementary Table S2. ^c^ The enrichment of these gene groups was tested by Fisher’s exact test (*p* < 0.05). Further data on the gene groups are available in Supplementary Table S3.

**Table 3:** Effect of betamethasone (BM) on the virulence attributes of *C. albicans*.

Virulence Attributes	QMY23 (WT)		cappz1 (KO)	
	Untreated	+BM	Untreated	+BM
Extracellular proteinase activity (Pz values) ^a^	0.72 ± 0.05	0.71 ± 0.06	0.74 ± 0.04	0.73 ± 0.05
Secreted phospholipase activity (Pz values) ^a^	0.48 ± 0.06	0.44 ± 0.07	0.45 ± 0.06	0.43± 0.08
Hypha formation (%) ^b^				
7 days	15.0 ± 3.1	17.5 ± 1.8	9.7 ± 2.4 ^c^	11.1 ± 2.1 ^c^
10 days	31.3 ± 3.3	33.2 ± 2.5	24.5 ± 2.1 ^c^	25.1 ± 2.9 ^c^

Mean ± SD for three independent experiments is presented. Cells were treated with 2 mM BM for 5 h. ^a^ Enzyme activities (Pz values) were determined by dividing the colony diameter and precipitation zones on egg yolk (EY) plates or clear areas on bovine serum albumin (BSA) media-plus-colony diameter after 5 days of incubation at 30 °C. ^b^ Hyphal growth % was calculated using the following formula: (width of hyphal ring)/(colony diameter + hyphal ring) × 100. ^c^ Significant differences (*p* < 0.05) between KO vs. WT.

**Table 4:** Betamethasone (BM) or/and menadione sodium bisulfite (MSB) induced changes in the antioxidant enzyme activities and redox homeostasis of *C. albicans*.

Oxidative Stress Related Parameters	QMY23 (WT)				cappz1 (KO)			
	Untreated	+BM	+MSB	+BM + MSB	Untreated	+BM	+MSB	+BM + MSB
glutathione reductase (mkat/kg protein)	1.8 ± 0.4	1.8 ± 0.45	2.5 ± 0.2 ^c^	3.2 ± 0.6 ^c^	2.3 ± 0.15 ^b^	2.1 ± 0.15	3.3 ± 0.25 ^b,c^	5.0 ±0.85 ^b,c,d^
glutathione peroxidase (mkat/kg protein)	0.17 ± 0.02	0.19 ± 0.01	0.22 ± 0.02 ^c^	0.26 ± 0.03 ^c^	0.22 ± 0.04 ^b^	0.21 ± 0.02	0.34 ± 0.07 ^b,c,e^	0.44 ± 0.05 ^b,c,d^
catalase (kat/kg protein)	0.44 ± 0.07	0.46 ± 0.06	0.65 ± 0.08 ^c^	0.83 ± 0.17 ^c^	0.6 ± 0.11	0.59 ± 0.10	0.9 ± 0.17 ^b,c^	1.7 ± 0.47 ^b,c,d^
superoxide dismutase (mU/mg protein)	52 ± 2.3	50.8 ± 1.4	56.9 ± 3.3	59.4 ± 3.1 ^c^	56.6 ± 2.3	54.5 ± 2.4	65.3 ± 4.5 ^c^	69.5 ± 7.3 ^c^
DCF (nmol DCF/OD_640_) ^a^	13.4 ± 3.1	12.5 ± 3.3	21.5 ± 2.0 ^c^	26.5 ± 2.6 ^c,d^	33.1 ± 2.3 ^b^	32.8 ± 3.0 ^b^	60.3 ± 8.7 ^b,c,e^	95.2 ± 11.9 ^b,c,d^

Samples were treated as in Figure 1. Mean ± SD values calculated from three independent experiments are presented. ^a^ Redox change was characterized by the 2′,7′-dichlorofluorescin diacetate (DCF) assay. Increased DCF production is an indicative of the redox imbalance. ^b^ Significant difference between untreated KO vs. untreated WT and treated KO vs. treated WT, ^c^ Significant difference between treated WT vs. untreated WT and treated KO vs. untreated KO. ^d^ Significant interaction between the effects of BM and MSB according to two-way ANOVA in the studied strain. ^e^ Significant interaction between the effects of MSB and the deletion of *CaPPZ1* according to two-way ANOVA (BM pre-treated cultures were omitted from the calculations).

## Data Availability

Original RNASeq data obtained in this work have been deposited in NCBI’s Gene Expression Omnibus [21] and are accessible through GEO Series accession number GSE173668 (https://www.ncbi.nlm.nih.gov/geo/query/acc.cgi?acc=GSE173668; accessed on 5 July 2021).

## Supplementary Materials

The following are available online at https://www.mdpi.com/article/10.3390/jof7070540/s1, Figure S1: Effect of BM and/or MSB on microbial growth, Figure S2: Principal component analysis of the transcriptome data, Table S1: Oligonucleotide primers used in RT-qPCR analysis, Table S2: Results of the gene set enrichment analysis, Table S3: Transcription data of selected gene groups, Table S4: Results of RT-qPCR experiments.
