# Supplementary material for: The Negative Effect of Protein Phosphatase Z1 Deletion on the Oxidative Stress Tolerance of Candida albicans Is Synergistic with Betamethasone Exposure
Source: J Fungi (Basel). 2021 Jul 6;7(7):540. doi: 10.3390/jof7070540 (PMC8305657; doi:10.3390/jof7070540)
Supplement: Supplementary file 1 [file jof-07-00540-s001.zip › jof-1267055-supplementary/FigureS1_Growth assay.pdf]

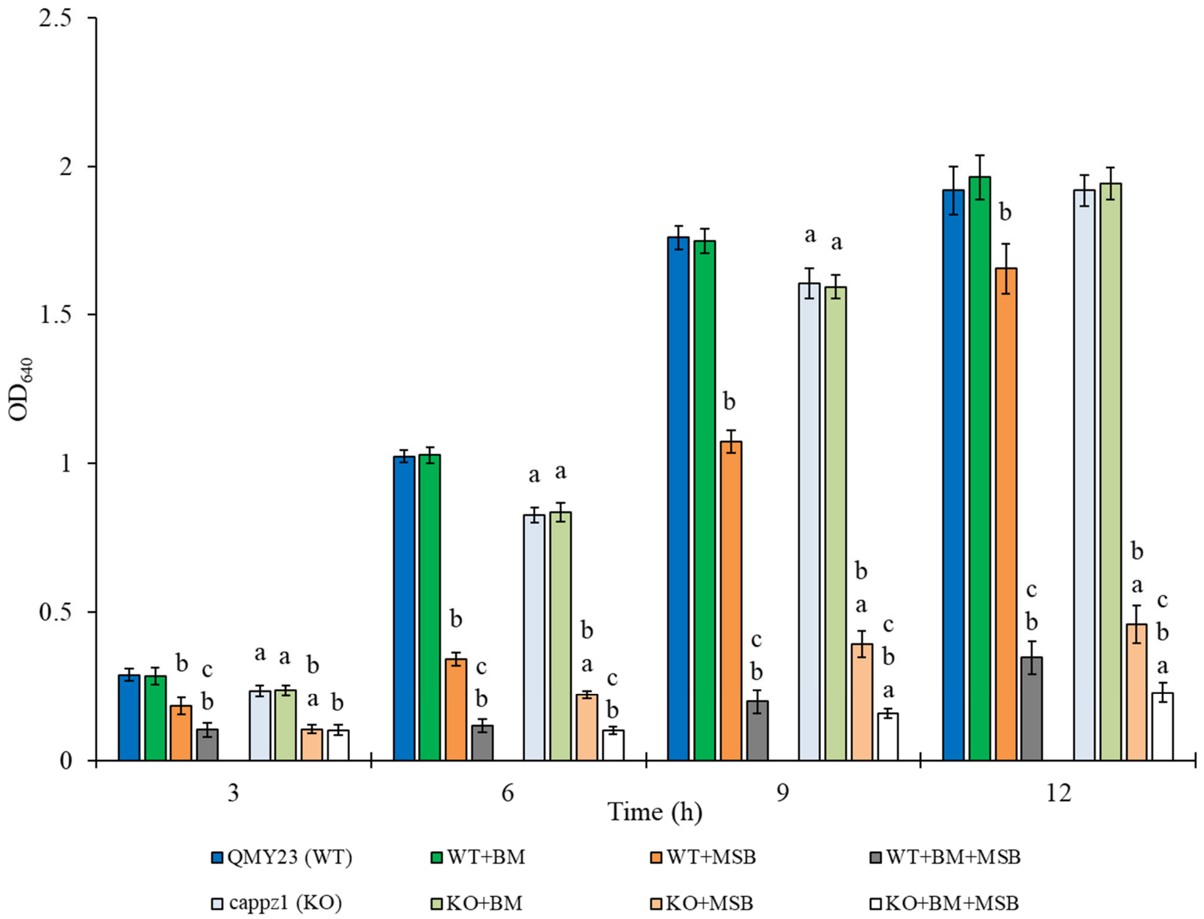

Supplementary Figure S1. Effect of BM and/or MSB on microbial growth. Growth of WT or KO in YPD broth with or without 12 h 2 mM BM treatment was monitored based on OD_{640} measurements. MSB was added at 0 h in 0.5 mM final concentration to half of the samples. Data represent mean ± SD values calculated from 3 independent experiments.

[a] Significant difference between untreated KO *vs* untreated WT and treated KO *vs* treated WT

[b] Significant difference between treated WT *vs* untreated WT and treated KO *vs* untreated KO

[c] Significant interaction between the effects of BM and MSB according to two-way ANOVA.
