# Supplementary material for: The Negative Effect of Protein Phosphatase Z1 Deletion on the Oxidative Stress Tolerance of Candida albicans Is Synergistic with Betamethasone Exposure
Source: J Fungi (Basel). 2021 Jul 6;7(7):540. doi: 10.3390/jof7070540 (PMC8305657; doi:10.3390/jof7070540)
Supplement: Supplementary file 1 [file jof-07-00540-s001.zip › jof-1267055-supplementary/FigureS2_PCA_New.pdf]

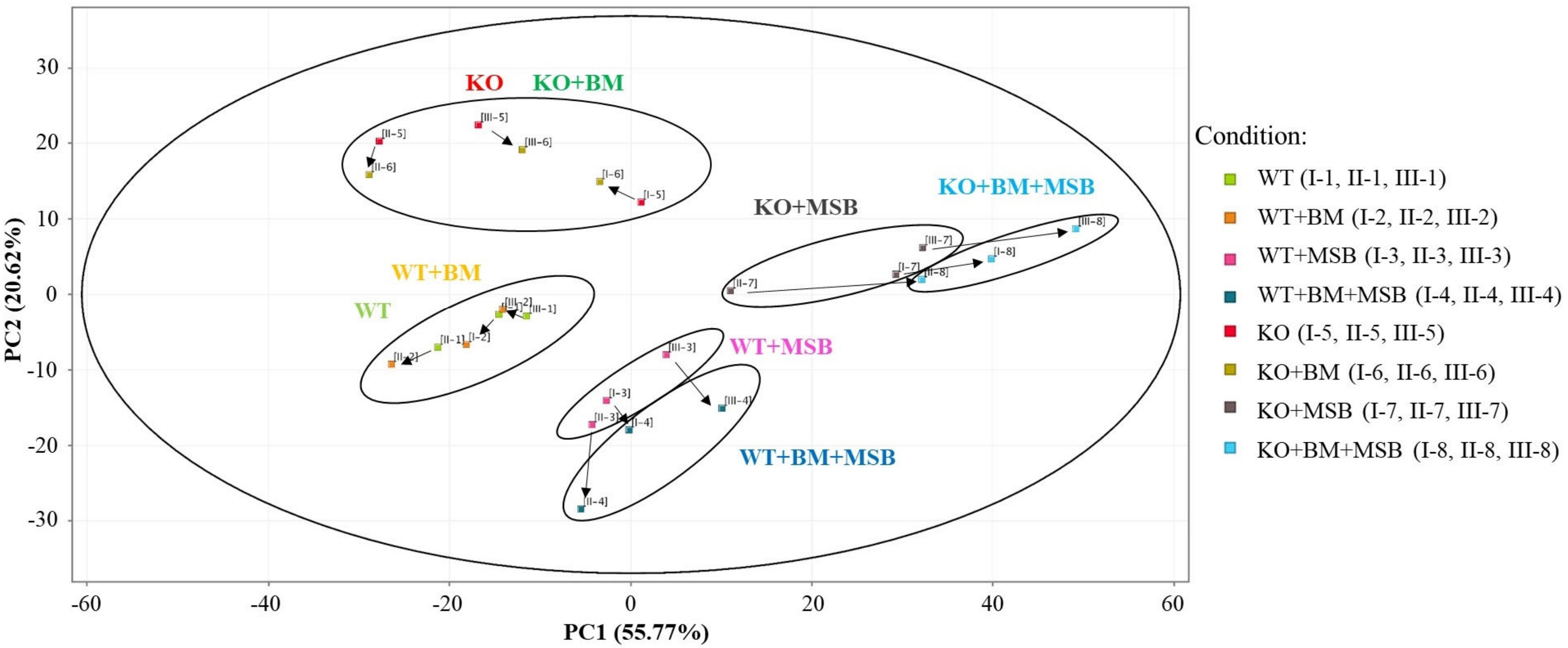

Supplementary Figure S2. Principal component analysis of the transcriptome data. Symbols represent untreated control (WT, KO), 2 mM betamethasone sodium phosphate (BM) or/and 1.5 mM menadione sodium bisulfite (MSB) treated cultures. The distribution of transcriptome data obtained in three independent series of experiments (I, II and III) under eight conditions (1-8) fall close to each other (circled) in the graph. Arrows represent the differences between two paired transcriptomes within one set of the three independent experiments. Note, that (i) KO is consistently

different from WT under all experimental conditions, (ii) BM had no significant influence on either WT or KO, but (iii) enhances the effect of MSB in both strains.
