# Supplementary material for: The Negative Effect of Protein Phosphatase Z1 Deletion on the Oxidative Stress Tolerance of Candida albicans Is Synergistic with Betamethasone Exposure
Source: J Fungi (Basel). 2021 Jul 6;7(7):540. doi: 10.3390/jof7070540 (PMC8305657; doi:10.3390/jof7070540)
Supplement: Supplementary file 1 [file jof-07-00540-s001.zip › jof-1267055-supplementary/TableS1_Primer list.pdf]

Supplementary Table S1. Oligonucleotide primers used in RT-qPCR analysis

cDNA name Gene / ID	Function	Forward primer (5'-3')	Reverse primer (5'-3')
**Controls**			
PPZ1 (CR_06420W)	Ser/Thr protein phosphatase	TCACAACAACGTCCACAACCA	GACAACGGTGAACCTGGC
ACT1 (C1_13700W)	Actin	GTTGCTCCAGAAGAACATCC	ACCATCACCAGAATCCAAAAC
RIP1 (C3_04430W)	Ubiquinol cytochrome c-reductase	ACAACCACCACCACCTTATC	AGCCATAGCCAAAACATCAG
EFG1 (CR_07890W)	Transcription factor	TTGCTGCTGCTACTACTACTG	CTGACCATACTGTTGAGGATAC
**Oxidative stress response**			
CAT1 (C1_06810W)	Catalase	TTTCTCTTTTGGGTGTGGATG	GGTGGTGAATTAGGTTCTGC
SOD1 (C4_02320C)	Superoxide dismutase	TTGAACAAGAATCCGAATCCG	GAAATGAGGACCAGCAGAAG
SOD3 (C7_00110W)	Superoxide dismutase	TGCTCAGTATGGGTCTGTTTC	ATCCTGATTGGCAGTAGTGAC
GLR1 (C5_01520C)	Glutathione reductase	TTTTCCGCTGACAAGACTTTG	ATTCAACCCCAATGTAACCAG
TRR1 (C5_02710W)	Thioredoxin reductase	CGAAGGTATGTTGGCTAATG	CTTTGGAGATGGTTTCAGTG
**Membrane transport**			
CDR1 (C3_05220W)	Drug transmembrane transporter	GTCGCTGGTGAAGGTTTG	AAGTGCTGATGGTTGATGG
MDR1 (C6_03170C)	Drug transmembrane transporter	TGCCCATCCAGAAGAAG	ACCATCCCAAGTGACAAC
PMA1 (C3_00720W)	Plasma membrane H(+)-ATPase	TGAAGACGAAGACATTGACC	CTAAACCAGCAGCCAAAAC
HGT17 (C4_01070W)	Hexose transmembrane transporter	GTGATTGCCGAGAAGGAAG	CAGCCGACATTTTCAAAGTC
**Metabolism**			
PCK1 (CR_00200W)	Gluconeogenesis	GTTGTTGACGCTTATGCTG	ACCTTTAGTGTGGATGTTGG
PXP2 (C3_01930W)	Fatty acid beta-oxidation	CGGTTCCTTTGGTATGAC	TGTGAGTGGCAGAATGAG
CFL4 (C5_01360W)	Ferric reductase	TGAGTTGGGTCTTTTTCGC	ACGGCTGTAGTTGTGTTTCG
FTR1 (C1_14130W)	Iron permease	TAGTCCAGCCACCTCTTTC	AACCAGCAGCAATCAAGTAC
